# Identification of four mitochondria-related genes in sepsis based on RNA sequencing technology

**DOI:** 10.1186/s12865-024-00623-1

**Published:** 2024-05-16

**Authors:** ShilinLi, Yingchun Hu

**Affiliations:** Department of Emergency Medicine, The Affiliated Hospital of Southwest Medical University, 25 Taiping Street, Jiangyang District, Luzhou, Sichuan China

**Keywords:** Sepsis, Mitochondria-associated gene, 10X single-cell sequencing technology (scRNA-seq)

## Abstract

**Objectives:**

The purpose of this study was to identify and analyze the mitochondrial genes associated with sepsis patients in order to elucidate the underlying mechanism of sepsis immunity and provide new ideas for the clinical treatment of sepsis.

**Methods:**

The hospitalized cases of sepsis (*n* = 20) and systemic inflammatory response syndrome (SIRS) (*n* = 12) admitted to the Emergency Intensive Care Unit (EICU) of the Affiliated Hospital of Southwest Medical University from January 2019 to December 2019 were collected consecutively. RNA-seq was used to sequence the RNA (mRNA) of peripheral blood cells. Bioinformatics techniques were used to screen and identify differentially expressed RNAs, with an absolute value of fold change (FC) greater than or equal to 1.2 and a false discovery rate (FDR) less than 0.05. At the same time, mitochondrial genes were obtained from the MitoCarta 3.0 database. Differential genes were then intersected with mitochondrial genes. The resulting crossover genes were subjected to GO, KEGG, and PPI analysis. Subsequently, the GSE65682 dataset was downloaded from the GEO database for survival analysis to assess the prognostic value of core genes, and GSE67652 was downloaded for ROC curve analysis to validate the diagnostic value of core genes. Finally, the localization of core genes was clarified through 10X single-cell sequencing.

**Results:**

The crossing of 314 sepsis differential genes and 1136 mitochondrial genes yielded 28 genes. GO and KEGG analysis showed that the crossover genes were mainly involved in the mitochondrion, mitochondrial matrix, and mitochondrial inner membrane. Survival analysis screened four genes that were significantly negatively associated with the prognosis of sepsis, namely *FIS1, FKBP8, GLRX5*, and *GUK1.* A comparison of peripheral blood RNA-seq results between the sepsis group and the SIRS group showed that the expression levels of these four genes were significantly decreased in the sepsis group compared to the SIRS group. ROC curve analysis based on GSE67652 indicates these four genes’ high sensitivity and specificity for sepsis detection. Additionally, single-cell RNA sequencing found that the core genes were mainly expressed in macrophages, T cells, and B cells.

**Conclusions:**

Mitochondria-related genes (*FIS1, FKBP8, GLRX5, GUK1*) were underexpressed in the sepsis group, negatively correlated with survival, and mainly distributed in immune cells. This finding may guide studying the immune-related mechanisms of sepsis. This study protocol was reviewed by the Ethics Committee of the Affiliated Hospital of Southwest Medical University (ethics number: KY2018029), the clinical trial registration number is ChiCTR1900021261, and the registration date is February 4, 2019.

## Preface

Sepsis is a progressive condition that ranges from the initial systemic inflammatory response syndrome (SIRS) to sepsis, severe sepsis, and septic shock with or without multi-organ dysfunction [1]. It can manifest as either a common infection or a complex clinical syndrome characterized by an intricate inflammatory response. Consequently, sepsis has emerged as a highly challenging, expensive, and resource-intensive condition in the field of clinical practice [2]. Over the past two decades, the use of SIRS-based definitions for sepsis has greatly standardized the inclusion and exclusion criteria for sepsis clinical trials, thereby enhancing the comparability of trial results. The adoption of SIRS definitions is aimed at early identification and diagnosis of sepsis, which facilitates communication in literature, medical conferences, and routine medical practice. In addition, SIRS criteria are easy to use at the bedside, as the required parameters are readily available [3]. Myeloid cells play a vital role in the elimination of pathogens. After the initial inflammatory phase, classically activated macrophages (M1) undergo reprogramming to another M2 phenotype [4]. This second stage contributes to secondary immunosuppression, called immune paralysis, which occurs during sepsis and counteracts the clearance of infectious substances, ultimately worsening the pathological condition.

At present, studies have found that the pathophysiological mechanism of the disease mainly includes mitochondrial metabolism disorders, changes in molecular expression levels, and increased apoptosis. The critical role of mitochondria in immune cells and immunity is now widely recognized [5]. In rodent models of sepsis, alterations in mitochondrial function in the heart, muscle, and liver have been described [6]. The mitochondria, a membrane-bound organelle found in the majority of eukaryotic cells, possess a dual-layered membrane and measure approximately 0.5 to 10 micrometers in diameter [7]. It serves as a cellular structure responsible for energy production. Specifically, mitochondria play a pivotal role in intracellular oxidative phosphorylation and the synthesis of adenosis triphosphate, which serves as a vital energy source for cellular activities [8]. Furthermore, mitochondria play a crucial role in various cellular processes, including cell differentiation, cell signaling, and programmed cell death (apoptosis), as well as exerting control over cell growth and the cell cycle [9]. The metabolic activities of mitochondria are intricately linked to the apoptotic process, with mitochondrial proteins within the mitochondrial membrane space participating in both caspase-dependent and caspase-independent apoptotic events. In addition, apoptosis signals from the cell membrane, cytoplasm, and nucleus are integrated into the mitochondria, initiating the endogenous signaling pathway of apoptosis. Considering that mitochondrial dysfunction is a risk factor for the development of sepsis, the identification of effective mitochondria-related biomarkers for diagnosing and prognosing patients with sepsis should be an encouraging research direction.

**Fig. 1:**
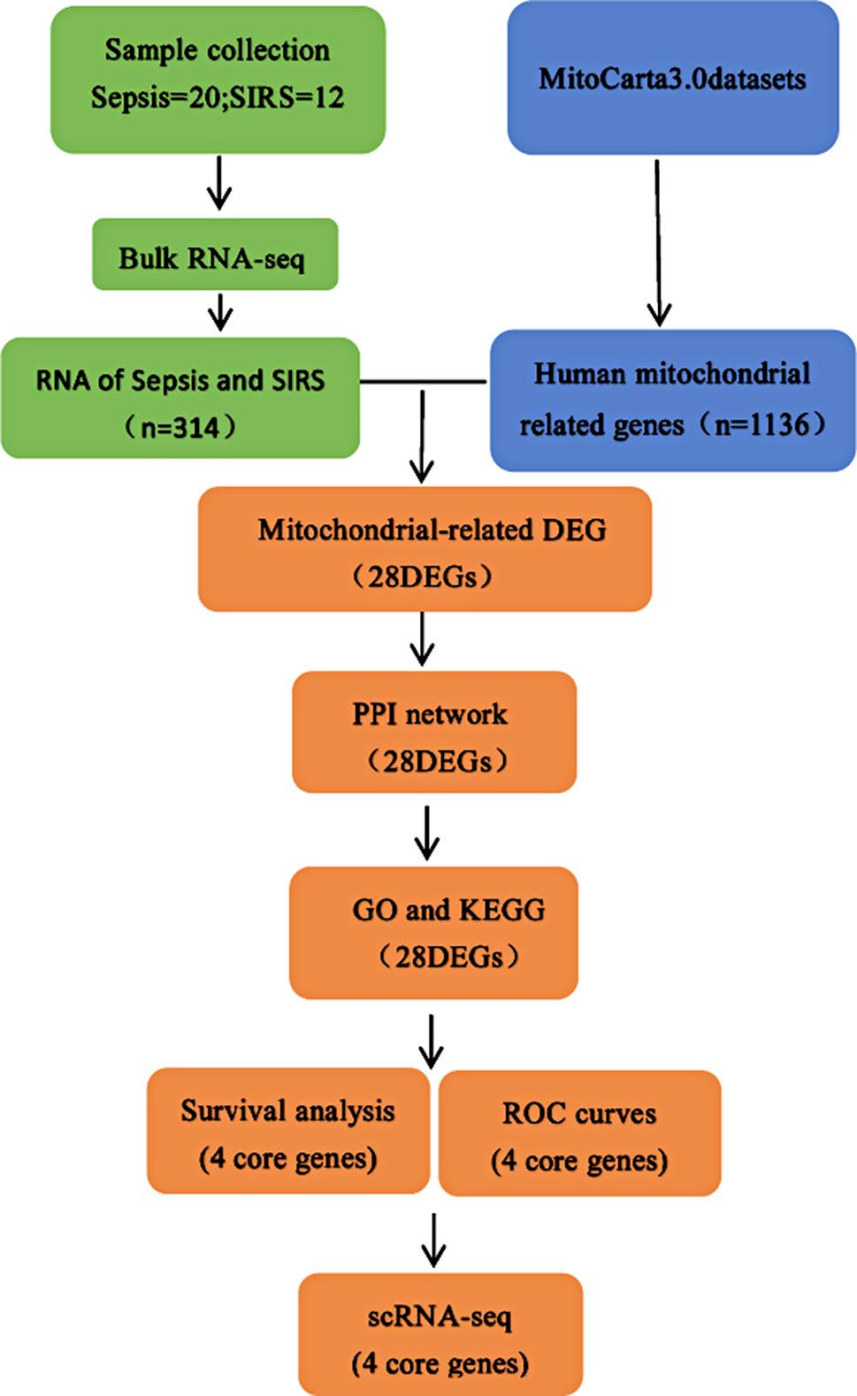
Flow chart of the experiment in this study. RNA-seq(RNA sequencing), DEGs(Differential genes), Gene Ontology (GO) analysis, Kyoto Encyclopedia of Genes and Genomes (KEGG) Analysis, Receiver Operating Characteristic (ROC) curve, scRNA-seq(Single-cell Sequencing)

In summary, this study utilized high-throughput sequencing technology to sequence the peripheral blood cells of 20 patients with sepsis and 12 patients with systemic inflammatory response syndrome. It also employed bioinformatics analysis methods for functional annotation and pathway analysis. The aim was to identify sepsis-related mitochondrial genes and locate them through single-cell sequencing. The findings of this study can guide further research on sepsis immunity-related mechanisms and offer new insights for the clinical treatment of sepsis (Fig. 1).

## Method

### Data collection

This study collected peripheral blood samples from 20 cases of sepsis and 12 cases of systemic inflammatory response syndrome at the EICU, Affiliated Hospital of Southwest Medical University, from January 2019 to December 2019. The inclusion criteria for sepsis cases were as follows: (1) meeting the diagnostic criteria for sepsis 3.0 (infection + ΔSOFA score ≥ 2) jointly published by the American Society of Critical Care Medicine (SCCM) and the European Society of Critical Care Medicine (ESICM) in 2016; (2) being aged ≥ 16 years old and ≤ 65 years old; (3) signing the informed consent form. Inclusion criteria for systemic inflammatory response syndrome are (1) body temperature > 38 °C or < 36 °C, (2) heart rate > 90 beats/minute, (3) respiratory rate > 20 breaths/minute or PaCO2 (arterial carbon dioxide partial pressure) < 32 mmHg, (4) white blood cell count > 12 × 10^9/L or < 4 × 10^9/L or immature granulocytes > 10%. The exclusion criteria were as follows: (1) individuals aged less than 16 years old or older than 65 years old; (2) patients with a history of organ failure; (3) patients with previous blood diseases, such as immunodeficiency; (4) patients who are unwilling to enroll. All subjects included in the study signed the informed consent form. The research protocol was reviewed by the Ethics Committee of the Affiliated Hospital of Southwest Medical University (ethics number: KY2018029), and the clinical trial registration number is ChiCTR1900021261.Studies involving human study participants are conducted by the Declaration of Helsinki [10].

### RNA sequencing

The collected samples are subjected to mRNA sequencing. Total RNA was extracted from peripheral blood cells using Trizol, and it was qualitatively and quantitatively assessed using the NanoDrop and Agilent 2100 bioanalyzer [11]. The raw data (mRNA) sequencing was filtered using the filtering software SOAPnuke (https://github.com/BGI-flexlab/SOAPnuke), and the resulting clean reads were saved in FASTQ format. Clean reads were then mapped to the reference genome using HISAT2 (v2.0.4). Then, fusion genes and differentially spliced genes (DSGs) were detected using Ericscript (v0.5.5) and rMATS (v3.2.5), respectively. Bowtie2 (v2.2.5) was used to align the clean reads with the gene set, and the gene expression level was calculated by RSEM (1.2.12) [12].

### Identification of differentially expressed genes

The online analysis platform IDEP0.96 (http://bioinformatics.sdstate.edu/idep/) was used to logarithmicize and normalize the data [13]. We used a boxplot of the density distribution to visualize the distribution of the two datasets, and a PCA plot to identify if there were differences between groups in the samples. The differential genes between the sepsis group and the SIRS group were screened, with the criteria being: absolute value of fold change (FC) ≥ 1.2 and false discovery rate (FDR) < 0.05. DESeq2 package in R was used for DEG analysis, and differential analysis results were visualized using a volcano plot to display DEGs [14].

### Screening of mitochondrial genes

MitoCarta3.0, an inventory of mammalian mitochondrial proteins and pathways developed by the Broad Institute, includes proteins encoded by 1,136 human and 1,140 mouse genes [15]. This updated version provides strong evidence for mitochondrial localization and includes submitochondrial chambers and pathway annotations. Therefore, we screened mitochondrial genes using the MitoCarta 3.0 database [16]. Enter the MitoCarta 3.0 database, enter “mitochondria” in the search box, select “Gene Product”, and limit the organism to “Homo sapiens” to download genes related to human mitochondria. Mitochondria-related genes that are closely associated with sepsis can be identified by cross-linking differential genes with mitochondria-related genes.

### Gene ontology (GO) analysis

Gene Ontology (GO) is a systematic approach utilized for the categorization of genes based on three fundamental aspects: biological process (BP), cellular component (CC), and molecular function (MF) [17]. To further explore whether crossover genes are involved in specific functions, the R package “cluster Profiler” was used to analyze crossover genes by Gene Ontology (GO), and a *p*-value of less than 0.05 was considered statistically significant.

### Kyoto encyclopedia of genes and genomes (KEGG) analysis

The KEGG (Kyoto Encyclopedia of Genes and Genomes) database is a comprehensive database that systematically analyzes gene functions and links genomic information with functional information [18]. It includes various databases such as the metabolic pathway database, hierarchical classification database, and genome database. Signaling pathways are the essence of basic scientific research, and this analysis paves the way for subsequent studies on signaling pathways. We conducted pathway enrichment analysis using the OmicShare tool, which is a free online platform for data analysis (www.omicshare.com/tools). Significantly enriched pathways in the DEGs were defined using hypergeometric tests compared to the genomic background. The calculated *p*-values were corrected for false discovery rate (FDR) with a threshold of FDR ≤ 0.05. Pathways meeting this criterion were identified as significantly enriched pathways in the cross-genome analysis [19].

### Protein-protein network interactions(PPI)

In functional genomics, although genes are the source of genetic information, proteins are the ultimate executors of gene functions and the main bearers of life activities. All important life activities require interactions between protein molecules or with other molecules. Protein-protein interactions (PPIs) are widely involved in the disease process [20]. In this study, to further screen the potential core genes, we submitted the crossover genes to the STRING (https://cn.string-db.org/) database. We selected “Homo sapiens” as the species option, “full STRING network” as the network type option, and “evidence” as the network edge meaning. Additionally, we set the correlation intensity factor to 0.15 and removed disconnected points in the network.

### Survival analysis

Survival analysis is a commonly used analytical method in biomedical research [21]. To explore the relationship between potential mitochondrial core genes screened by the PPI method and the prognosis of sepsis patients, we downloaded the public dataset GSE65682 from the GEO (Home-GEO-NCBI (nih.gov)) database [22]. The dataset was submitted by Scicluna BP et al. in 2015. It includes genetic data related to more than 400 patients with sepsis and a 28-day prognosis. We extracted relevant data and used Graphpad Prism 9.0 for graph analysis and a log-rank test for statistics. A *P*-value of less than 0.05 was defined as statistically significant. We validated the expression of four core genes based on peripheral blood RNA in the sepsis group (*n* = 20) and the SIRS group (*n* = 12).

### Receiver operating characteristic (ROC) curve

To evaluate the accuracy of diagnosing sepsis, this study focused on four core genes (*FIS1, FKBP8, GLRX5, GUK1*) and used ROC curves for verification. The ROC curve plots the area under the curve (AUC), where a higher AUC score indicates a better ability to distinguish between patients with and without the disease. Our study utilized the dataset from the GEO database GSE67652, which includes 12 healthy volunteers and 12 sepsis patients [23]. By comparing gene expression in septic patients to healthy volunteers in their admission samples, we analyzed gene expression. The ROC curve analysis was performed using MedCalc software.

### 10 X single-cell sequencing technology (scRNA-seq)

Single-cell genome sequencing can reveal the cellular location of target genes in tissues, as well as their function and characteristics at different stages and in different aspects [24]. In this study, 10X single-cell sequencing technology was used to explore the localization of each target gene in the cell lineage. We collected five blood samples (NC (normal controls) = 2; sepsis = 2; SIRS = 1) and pooled them. PBMCs were isolated by density gradient centrifugation for 10X Genomics, and the raw reads generated in high-throughput sequencing were sequenced in FASTQ format. CellRanger software was used for mass analysis. Further quality control of the data was performed using the Seurat software package15. PCA dimensionality reduction was performed using gene expression, and the results were then visualized by t-SNE. In addition, the FindAMarkers function is used to identify genetic biomarkers. Cellular localization of specific target genes facilitates the selection of specific cell lineages for later in vitro functional studies.

### Statistical analysis

The raw data from RNA-seq sequencing was logarithmically converted and compared. The log-rank test was used for the survival curve, and core gene validation was performed using an independent samples t-test. GraphPad Prism software 9.0 (GraphPad Inc., La Jolla, CA) was used to analyze the data and plot graphs.

## Results

### Population statistics and clinical characteristics

A total of 20 patients with sepsis and 12 patients with systemic inflammatory response syndrome were included in this project. Demographic and clinical characteristics of participants included sex, age, alanine aminotransferase, total bilirubin, creatinine, Hypersensitive troponin, white blood cell count, neutrophil count, monocyte count, lymphocyte count, thrombin time, international normalized ratio, and procalcitonin level (PCT), with continuous variables expressed as mean ± standard deviation, statistically analyzed using unpaired T-test (Table 1). The results showed that the inflammatory indexes of sepsis patients were increased, and there were statistically significant differences in total bilirubin, monocytes, and PCT.

**Table 1:** Demographic and clinical data of subjects (m ± sd). Gender, 28-day Finale (S: survivor, D: death), age, ALT, TBIL, creatinine(Crea), cardiac troponin (cTnI), Total white blood cell (WBC) count, neutrophil count, monocytes(MONO), lymphocyte(LY), thrombin time(TT), international normalized ratio, (INR) and procalcitonin(PCT)

Clinic items	Sepsis(*n* = 20)	SIRS(*n* = 12)	*P*
Gender(F/M) 28- Day Finale(S/D)	(13/7) (15/5)	(7/5) (2/10)	- -
Age(years)	55.00 ± 12.18	46.41 ± 14.27	0.51
ALT (U/L)	117.81 ± 210.39	57.27 ± 63.25	0.249
TBIL (umol/L)	21.07 ± 17.63	14.05 ± 4.89	0.037
Crea (umol/L)	167.75 ± 169.10	85.88 ± 60.95	0.078
CTnl (µg/L)	0.33 ± 0.20	0.80 ± 0.14	0.121
WBC (10^9/L)	13.72 ± 9.61	11.45 ± 5.27	0.50
NEUT (10^9/L)	11.76 ± 9.84	8.60 ± 4.82	0.62
MONO (10^9/L)	1.20 ± 2.17	0.63 ± 0.37	0.063
LY (10^9/L)	3.80 ± 6.30	0.94 ± 0.43	0.002
TT (min)	15.57 ± 1.72	15.50 ± 1.97	0.045
INR (s)	1.23 ± 0.17	1.20 ± 0.13	0.164
PCT (ng /ml)	40.39 ± 39.63	11.69 ± 25.64	0.007

### Differential mRNA identification

**Fig. 2:**
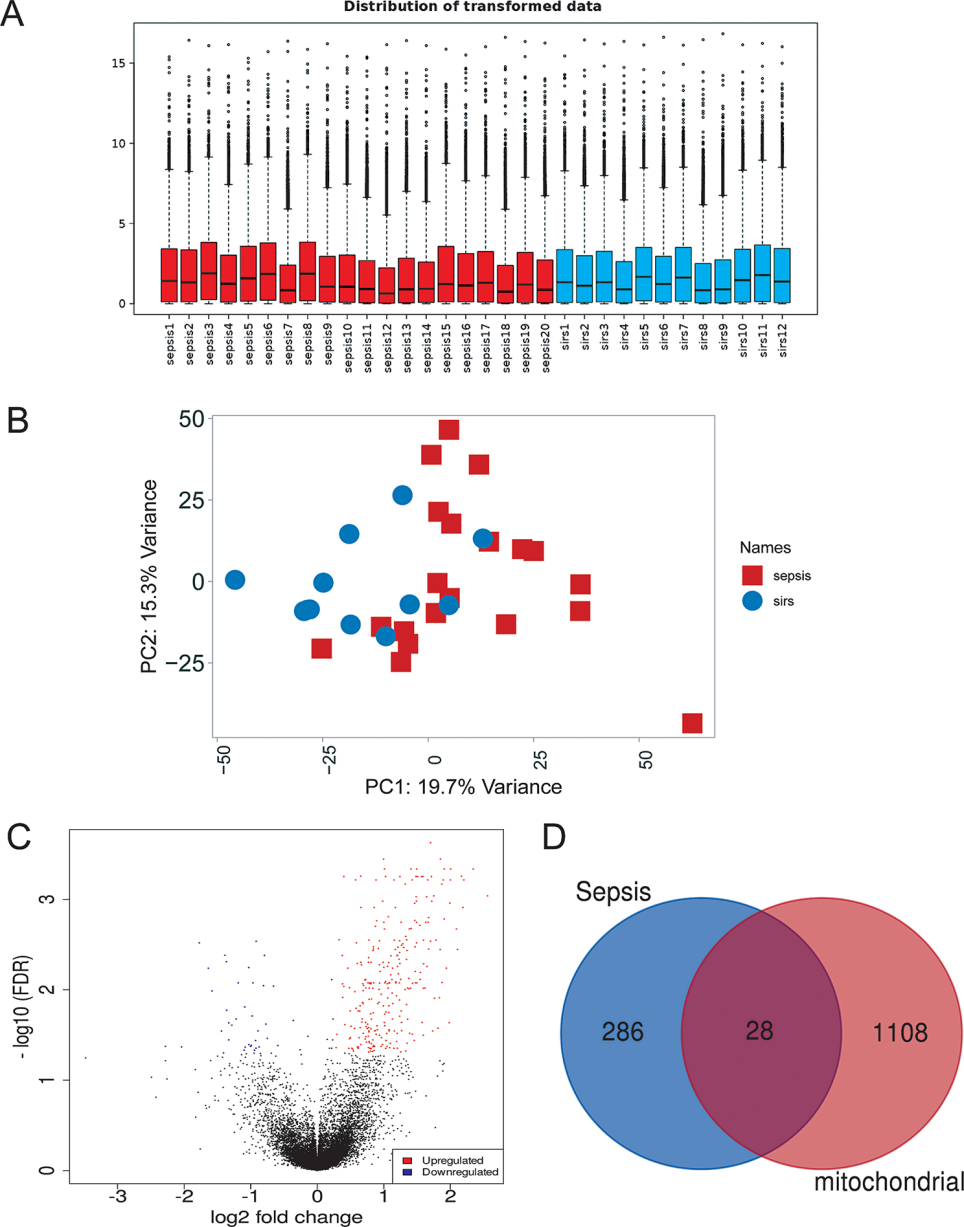
Bioinformatics analysis of RNA-seq data. (**A**)Data distribution after conversion: 20,717 genes in 32 samples. 14,875 were converted to Ensemble gene IDs in our database. The remaining 491 genes were saved in the data using the original ID.(**B**) Principal component analysis (PCA) showed that there were differences between our sepsis and SIRS groups. (**C**) Volcano map of differential genes, the selection criteria for differentially expressed genes were set to ≥ 1.2 and the error discovery rate (FDR) was < 0.05. (**D**) Wayne diagram, blue represents the sepsis differential gene (*n* = 314), red represents the human mitochondrial gene (*n* = 1136), and 28 sepsis-related mitochondrial genes were screened

The homogeneity and comparability of the two datasets after transformation were displayed through box plots showing the distribution of the data (Fig. 2A). The RNA-seq data underwent PCA analysis, which revealed the clear separation of all samples into two groups (Fig. 2B). A total of 314 DEmRNAs were selected under the conditions of absolute value of fold change (FC) ≥ 1.2 and false discovery rate (FDR) < 0.05, with 277 upregulated and 37 downregulated (Fig. 2C).

### Screening of mitochondria-related genes

A total of 1136 human mitochondrial genes were obtained from the MitoCarta 3.0 database. Analysis revealed an intersection of 1136 mitochondria-related genes with 314 genes exhibiting differential expression in sepsis, identifying 28 mitochondrial genes potentially associated with sepsis (Fig. 2D).

### GO

**Fig. 3:**
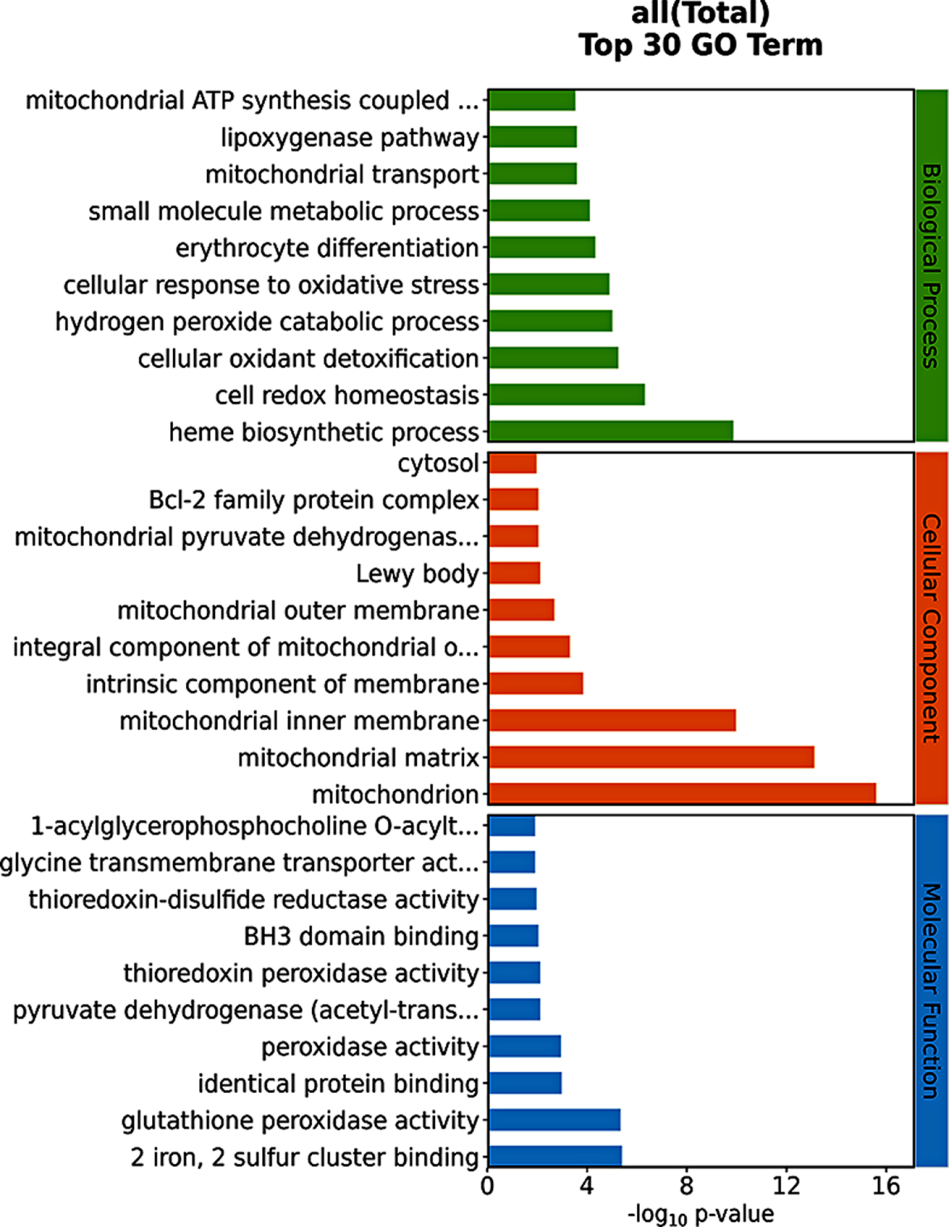
Gene Ontology (GO) analysis. The three levels of BP(biological process), CC(cellular component), and MF (molecular function)of GO analysis are represented from top to bottom, respectively. The length of the histogram indicates the number of genes

GO functional enrichment analysis showed that these crossover genes were mainly involved in biological processes such as heme biosynthetic process, cell redox homeostasis, and cellular oxidant detoxification. The enrichment of cell components associated with crossover genes showed changes in the mitochondrion, mitochondrial matrix, and mitochondrial inner membrane. In addition, the molecular functions associated with crossover genes mainly involve 2 iron, 2 sulfur cluster binding, glutathione peroxidase activity, and identical protein binding (Fig. 3).

### KEGG

**Fig. 4:**
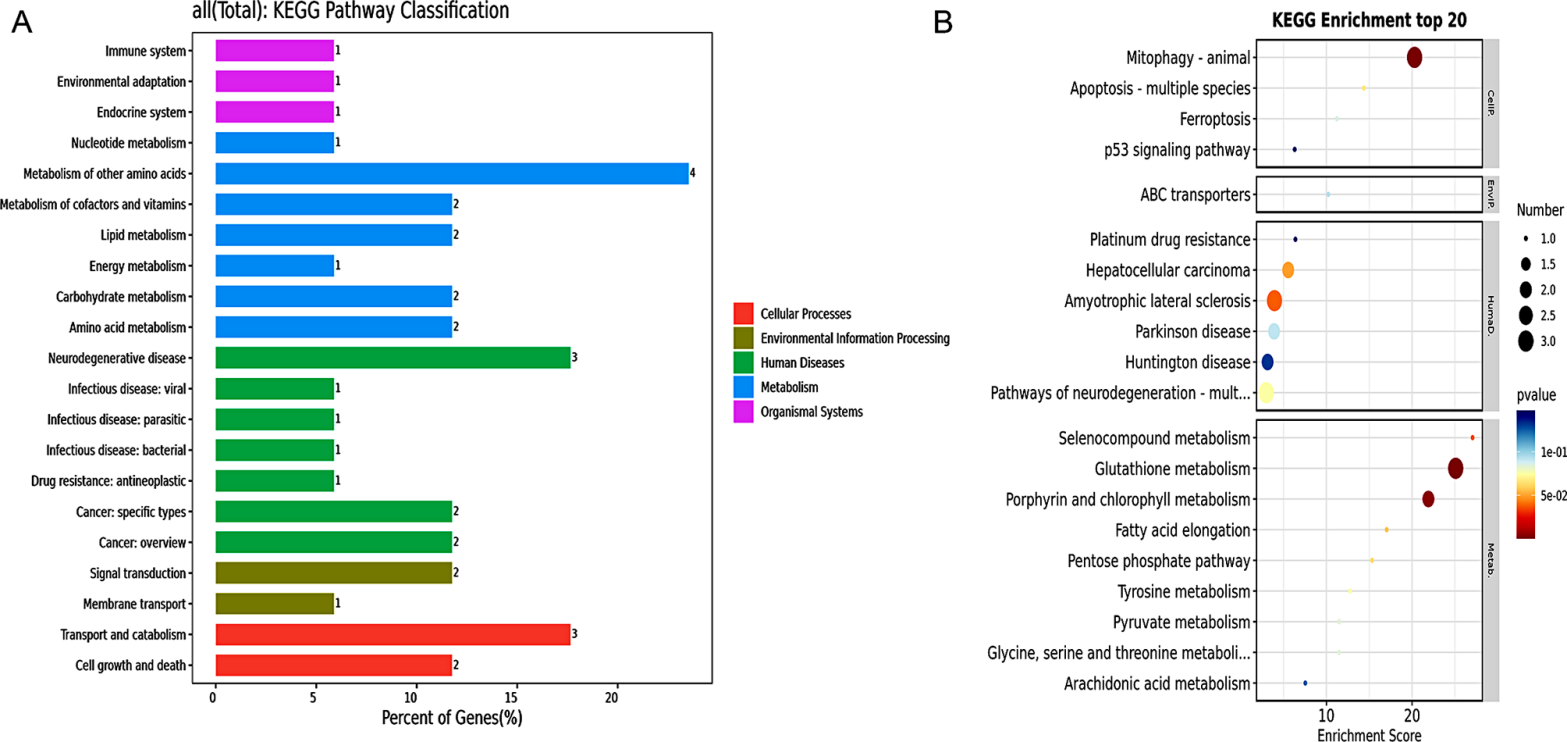
Kyoto Encyclopedia of Genes and Genomes(KEGG). (**A**) According to the analysis done by KEGG, there are 34 significant items. The right-hand side of the chart shows the enrichment A-level classification name. On the X-axis, one can observe the number of genes corresponding to each category, while on the Y-axis, the names of the B-level categories are displayed under each A-level category. The items on the chart include 14 metabolics, 3 environmental information processing, 5 cellular processes, 3 organism systems, and 11 human diseases. (**B**) Among the pathways with the highest significance in cross-gene terms, there are Mitophagy, Glutathione metabolism, and Porphyrin and chlorophyll metabolism. In the figure, the red color indicates a higher *P*-value, the blue color indicates a lower P-value, and the size of the circles represents the number of genes

The analyses carried out by KEGG identified 34 of the most noteworthy items, including 14 metabolisms, 3 environmental information processing, 5 cellular processes, 3 organism systems, and 11 human diseases (Fig. 4A). Among the most significant pathways of crossover genes are Mitophagy, Glutathione metabolism, and Porphyrin and chlorophyll metabolism (Fig. 4B).

### PPI

**Fig. 5:**
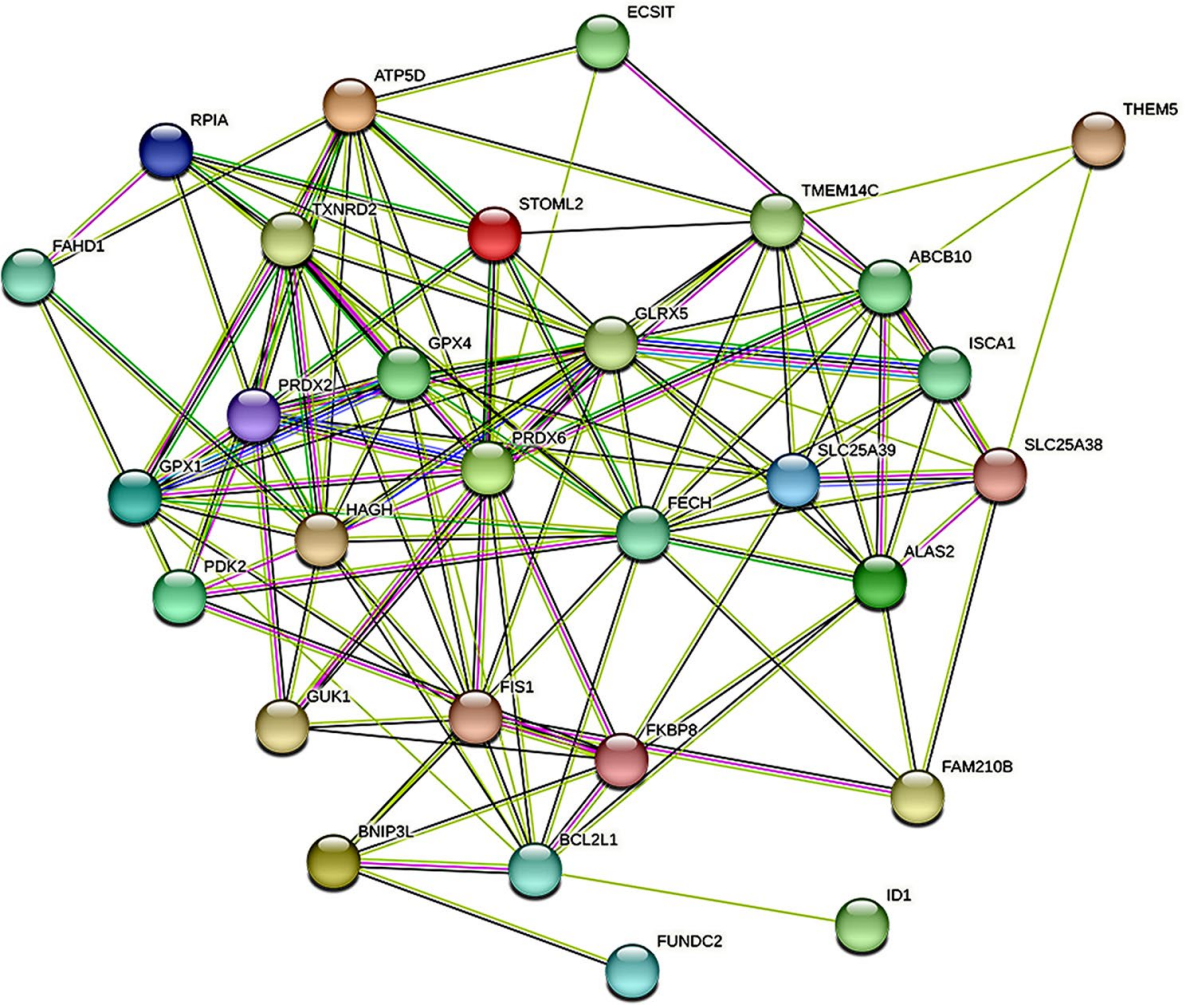
Protein-protein interaction (PPI) network. Based on the STRING protein interaction network diagram, multiple Intersection genes are located at the core of the network, and the more lines between each other, the closer the protein is related to the protein

In this study, protein-protein interaction analysis revealed that *ATP50, TXNRD2, STOML2, GPX4, GUK1, FIS1, FKBP8, GPX1, GLRX5*, and other genes were located in the central region of the PPI network (Fig. 5). These proteins have the potential to serve as core targets that can influence the prognosis of sepsis.

### Survival analysis

**Fig. 6:**
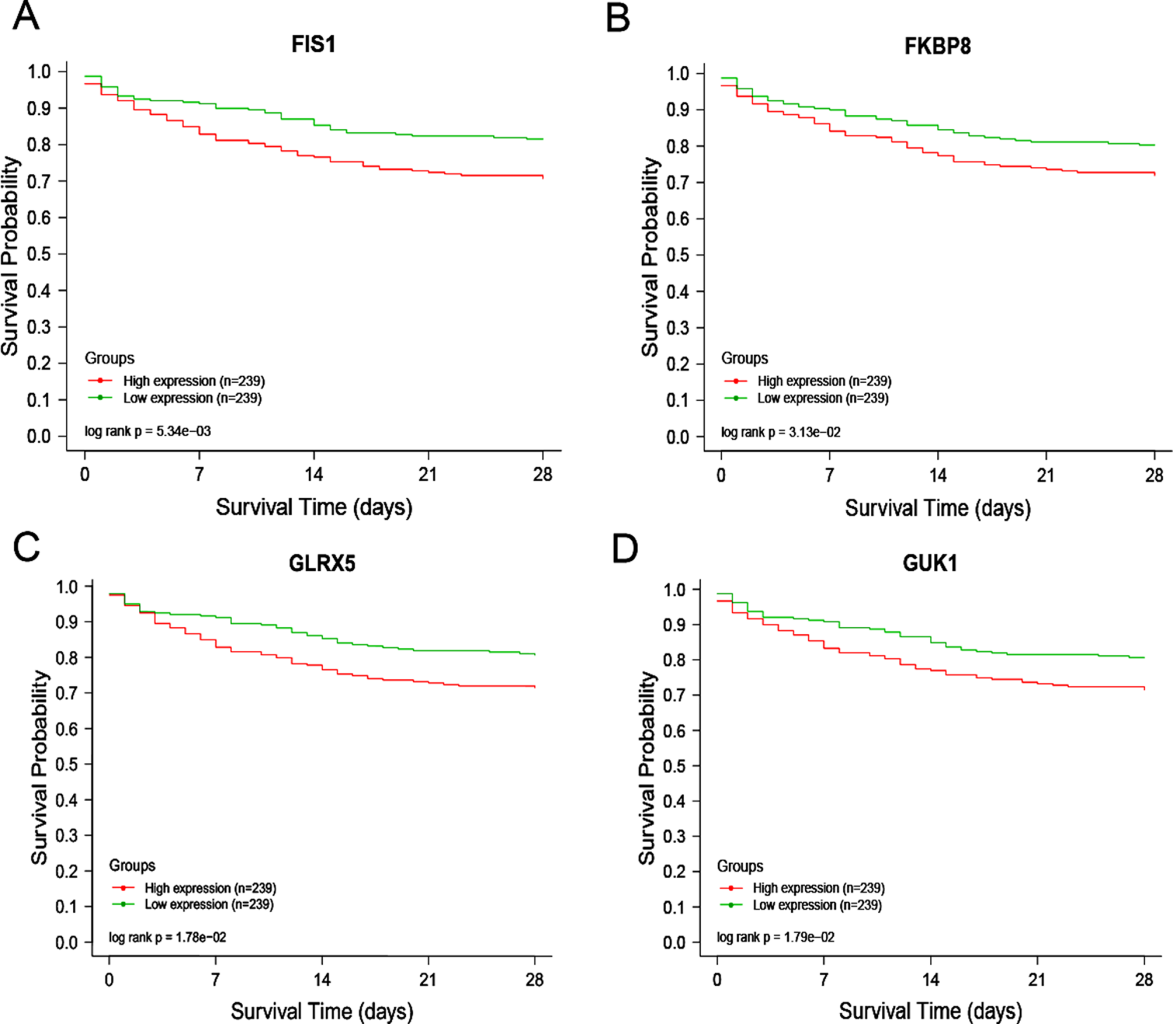
Clinical significance of core genes. Based on the GSE65682 dataset, all Intersection genes were divided into two parts according to the expression value, and the red line represented the high expression group (*n* = 239). The green line indicates the low expression group (*n* = 239); The abscissa is the recording time and the abscissa is the immediate survival rate. (**A**-**D**) indicated that low-expression *FIS1, FKBP8, GLRX5*, and *GUK1* were positively correlated with sepsis survival

**Fig. 7:**
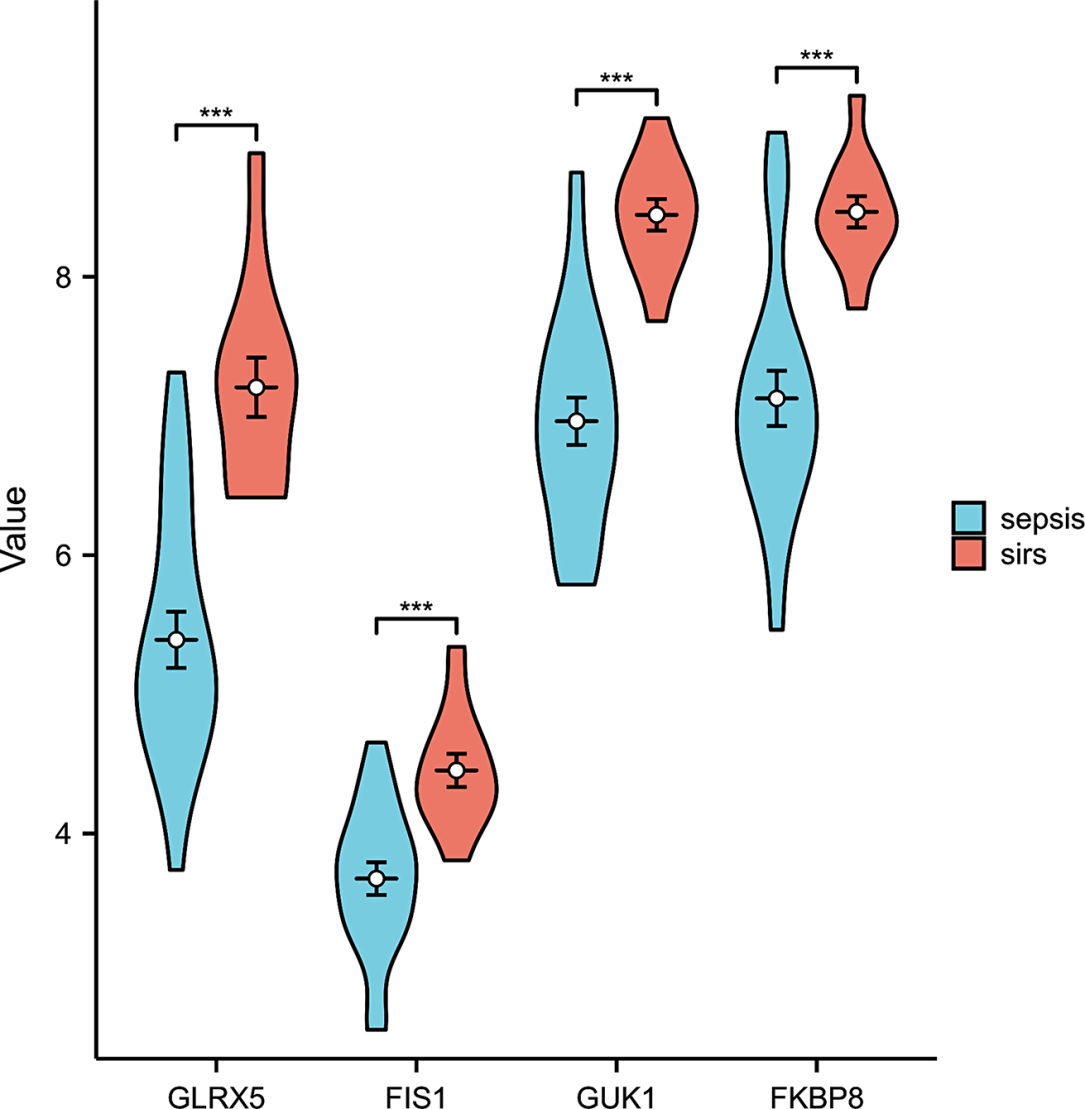
Core gene verification. Validation was based on peripheral blood sequencing data in the sepsis group (*n* = 20) and SIRS group (*n* = 12). The abscissa (x-axis) shows the four core genes, and the ordinate (y-axis) indicates the expression level of the core genes in the two groups. The results showed that the expression of *FIS1, FKBP8, GLRX5*, and *GUK1* at transcriptional levels was significantly higher in the SIRS group (****: *p* < 0.0001; ***:*p* < 0.001; **: *p* < 0.01; *: *p* < 0.05)

Based on the survival analysis of the GSE65682 dataset in the GEO database, we observed that the 28-day survival rate of patients with low expression of *FIS1, FKBP8, GLRX5*, and *GUK1* was higher than that of the high expression group (*P* < 0.05) (Fig. 6A-D). This finding suggests that these genes are inversely correlated with the prognosis of sepsis patients, and their low expression levels may become a new focus in sepsis research. Therefore, these findings provide valuable insights into the development of mitochondria-targeted therapy for sepsis. We conducted RNA sequencing on peripheral blood samples collected from 20 patients with sepsis and 12 patients with SIRS. We compared the gene expression levels of both groups and found that *FIS1, FKBP8, GLRX5*, and *GUK1* were underexpressed in the sepsis group but overexpressed in the SIRS group. This difference in gene expression was found to be statistically significant (Fig. 7).

### Receiver operating characteristic (ROC) curve

**Fig. 8:**
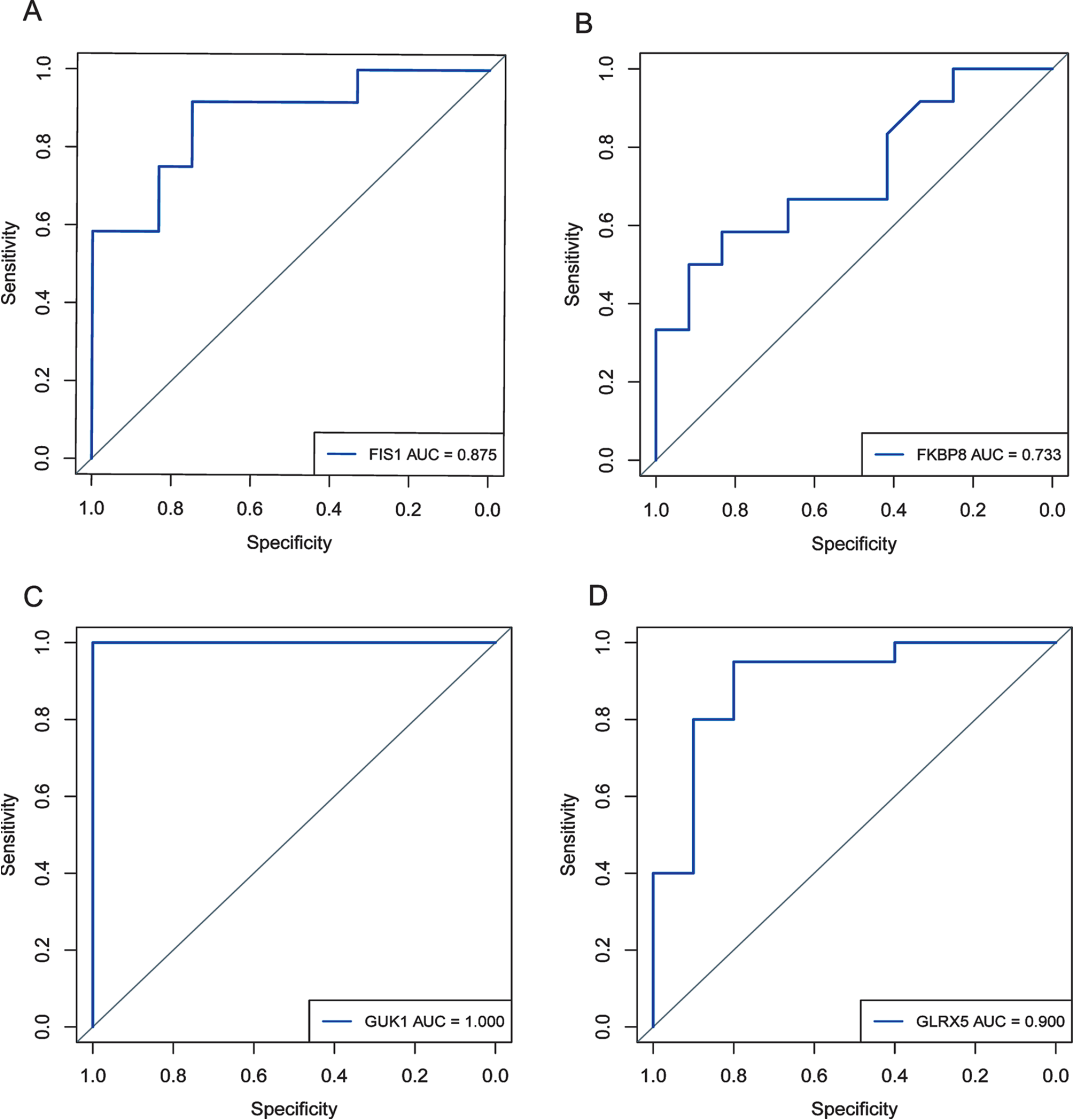
Receiver Operating Characteristic (ROC) Curve. (**A**-**D**) Based on the ROC curve of the GSE67652 dataset, the AUC values for *FIS1* (0.875), *FKBP8* (0.733), *GUK1* (1.00), and *GLRX5* (0.900) demonstrate that mitochondrial-related genes exhibit high sensitivity and specificity for sepsis

ROC curve analysis showed that the AUC values of *FIS1, FKBP8, GLRX5*, and *GUK1* were respectively as follows: *FIS1* (0.875), *FKBP8* (0.733), *GUK1* (1.00), and *GLRX5* (0.900) (Fig. 8A-D). The high sensitivity and specificity of these core genes to sepsis were indicated, therefore, this finding provides valuable insights for the development of mitochondria-targeted therapy for septicemia.

### 10 X single-cell sequencing technology

**Fig. 9:**
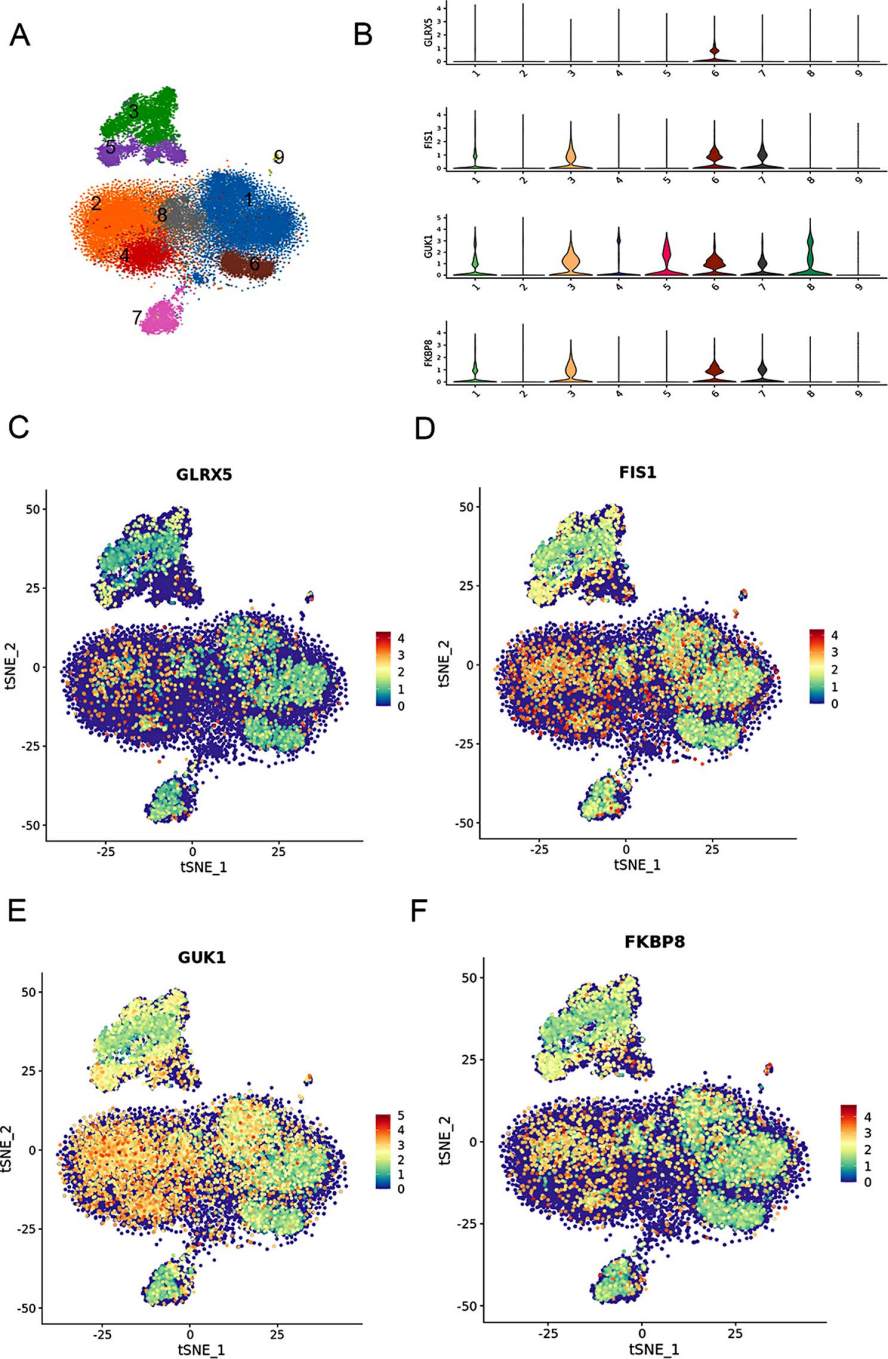
Single-cell sequencing localization. (**A**-**B**) General sequencing of mixed samples. After descending clustering, the cells were divided into 9 categories, and the cell types identified by marker genes were B cells, NK cells, T cells, platelets, and monocytes-macrophages. 3 and 5 represent monocytes-macrophages, 4 represent NK cells, 1, 2, 6, and 8 represent T cells, 7 represent B cells, and 9 represent platelets. (**C**-**F**) suggests that the core genes *GLRX5, FIS1, GUK1*, and *FKBP8* are mainly localized to 1, 3, 5, 6, 7, and 8 cell populations, i.e., monocyte-macrophage cell lines, B cells, and T cells

A total of five single-cell transcriptome sequencing samples were completed in this analysis. The number of qualified cells after culling double-celled, multicellular, and apoptotic cells in the sample ranged from 6,000 to 12,000. After performing hierarchical clustering, the cells were divided into nine categories. The identified cell types, determined by marker genes, included B cells, NK cells, T cells, platelets, and monocytes-macrophages. 3 and 5 of these represent monocytes-macrophages, 4 represent NK cells, 1, 2, 6, and 8 represent T cells, 7 represent B cells, and 9 represent platelets (Fig. 9A B). *GLRX5* is predominantly located in the 6th cell population, the T cell population (Fig. 9C). *FIS1* is predominantly localized to the 3rd, 6th, and 7th cell populations, i.e., monocyte-macrophages, T cells, and B cell populations (Fig. 9D).*GUK1* is predominantly localized to the 1, 3, 5, 6, 7, and 8 cell populations, i.e., monocyte-macrophages, T cells, and B cell populations (Fig. 9E). *FKBP8* is predominantly localized to the 3, 6, and 7 cell populations, i.e., monocytes-macrophages, T cells, and B cells (Fig. 9F).

## Discussion

Sepsis is a highly inflammatory disease that can lead to septic shock and multi-organ dysfunction [25]. Despite the in-depth understanding of the pathogenesis of sepsis, there has been no significant improvement in the mortality rate of sepsis in clinical treatment at home and abroad. Therefore, early diagnosis and treatment are essential to prevent serious complications. Currently, there are many biomarkers used in the prediction of sepsis prognosis, such as CD247, FYN, CD96, etc., most of which have been studied individually as single biomarkers [26]. There is an increasing amount of evidence indicating that multi-target approaches are more effective in treating complex diseases such as sepsis, cancer, and GVHD than single-target methods [27]. Therefore, there is a great need to find more effective biomarkers for targeted therapy and prognostic prediction. In the sepsis state, mitochondrial activity becomes unbalanced, morphology changes significantly, and function is significantly inhibited. Electron microscopy has observed ultrastructural destruction in the mitochondria of important organs such as the liver, kidney, heart, lungs, brain, and others isolated from animal models of sepsis. These changes include mitochondrial swelling, crest disappearance, mitochondrial vacuole formation, matrix destruction, membrane rupture, and other alterations, accompanied by mitochondrial deformation and shrinkage [28]. Simultaneously, the state of sepsis is accompanied by impaired physiological functioning of mitochondria, leading to alterations such as oxidative stress, calcium overload, and diminished biological functionality. Given the pivotal role of mitochondria in cellular proliferation and apoptosis, they serve as noteworthy pharmacological targets [29]. In this study, we identified for the first time that four mitochondrial genes (*FIS1, FKBP8, GUK1, GLRX5*) can significantly impact the prognosis of sepsis patients, with high sensitivity and specificity for sepsis. We collected 5 peripheral blood samples for single-cell sequencing. Ultimately, through single-cell sequencing, we found that the transcription levels of these four genes are mainly located in immune cells. We believe they have the potential to become biomarkers. The findings of this study provide valuable insights for the development of targeted therapies aimed at mitochondrial interventions.

Mitochondrial fission 1 protein (*FIS1*) is a member of the *FIS1* family [30]. *FIS1* is a single-pass membrane protein containing a TPR repeat. *FIS1* is a component of the mitochondrial complex that promotes mitochondrial fission. *FIS1* triggers the release of cytochrome C from the mitochondria into the cytoplasm, ultimately resulting in apoptosis. In addition, *FIS1* is involved in the growth and division of peroxisomes. The C-terminus of *FIS1* is required for mitochondrial or peroxisome localization, while the N-terminus is required for mitochondrial or peroxisome fission, localization, and regulation of interactions with DNM1L [31]. In this study, we found that *FIS1* was downregulated in the peripheral blood transcriptome results of sepsis, with statistically significant differences. Using the GEO database and downloading GSE65682 for 28-day survival analysis, we found that sepsis patients with low expression of *FIS1* had a higher 28-day survival rate compared to the high expression group (*P* < 0.05). This finding suggests that these genes are negatively correlated with the prognosis of sepsis patients. Using the GSE67652 dataset for ROC curve analysis, *FIS1* had an AUC score of 0.875. Based on this, we hypothesize that the low expression level of *FIS1* may become a new focus in sepsis research, and it shows high sensitivity and specificity for sepsis. Finally, we collected 5 peripheral blood samples for single-cell sequencing and found that *FIS1* is mainly located within immune-related cells. Our findings provide new information to guide further studies on mitochondrial genes and their mechanisms of action.

The protein encoded by this gene, *GUK1*, is an enzyme that catalyzes the transfer of phosphate groups from ATP to guanosine monophosphate (GMP) to form guanosine diphosphate (GDP) [32]. The encoded protein is considered a promising target for cancer chemotherapy. Several transcript variants encoding different isoforms have been found for this gene. In this study, we screened the key gene *GUK1* using bioinformatics methods. Survival analysis showed that low expression of *GUK1* in patients with sepsis could prolong their survival time. The ROC curve results show that both its sensitivity and specificity are relatively high. Additionally, single-cell sequencing mapping revealed that *GUK1* was mainly located in macrophages and immune cells. Therefore, we speculate that low expression of *GUK1* is beneficial for the survival of patients with sepsis, and follow-up studies can use this as a starting point.

*FKBP8* (FK506-binding protein 8) is a protein that belongs to the immunophilic protein family [33]. It plays a crucial role in immune regulation and essential cellular processes, including protein folding and trafficking. Unlike other family members, this encoded protein does not appear to have PPIase activity. It may play a role in neurons associated with memory function. This study found that *FKBP8* is located at the core of the protein-protein interaction network, and survival analysis showed that patients with low expression had a higher 28-day survival rate. To validate the accuracy of the data, we downloaded the dataset GSE67652 to draw ROC curves and found that *FKBP8* had an AUC score of 0.733. Finally, single-cell sequencing localized *FKBP8* mainly in immune-related cells. Therefore, we speculate that *FKBP8* can serve as an innovative target for mitochondrial therapy, especially in terms of immune therapy, thus providing a new avenue for the clinical treatment of sepsis.

*GLRX5* encodes an evolutionarily conserved mitochondrial protein that is involved in the biogenesis of iron-sulfur clusters [34]. These clusters are required for maintaining normal iron homeostasis. Mutations in the *GLRX5* gene are associated with autosomal recessive pyridoxine-refractory sideroblastic anemia. In this study, we found that the low expression of *GLRX5* in sepsis patients is associated with prognosis. The accuracy of previous data was confirmed by ROC curve analysis. Single-cell sequencing revealed that this gene is mainly expressed in macrophages, T cells, and B cells. This study guides research on the immune-related mechanisms of sepsis.

## Conclusion

In summary, in this study, we used peripheral blood RNA sequencing of patients with sepsis and systemic inflammatory response syndrome to obtain human mitochondrial genes from the MitoCarta 3.0 database. We also obtained mitochondria-related genes that can significantly affect the prognosis of patients with sepsis through bioinformatics methods. Our study provides new information to guide subsequent functional studies of mitochondrial genes and their mechanisms of action. However, our research is limited by the small number of sequencing samples, which may lead to false positive results. In addition, the inference is based solely on sequencing data, lacking sufficient feasibility, and should be further validated for its mechanism.

## Data Availability

We intend to share individual deidentified participant data. Peripheral blood RNA sequencing data from 20 patients with sepsis and 12 SIRS are available in the China National GeneBank DataBase (CNGBdb) and can be found below:db.cngb.org/, under the accession: CNP0002611, you can access it now and it’s valid forever.
